# Targeting NAD metabolism regulates extracellular adenosine levels to improve the cytotoxicity of CD8+ effector T cells in the tumor microenvironment of gastric cancer

**DOI:** 10.1007/s00432-022-04124-9

**Published:** 2022-07-01

**Authors:** Han-Yuan Liu, Fu-Hui Wang, Jian-Ming Liang, Yuan-Yuan Xiang, Shu-Hao Liu, Shi-Wei Zhang, Cheng-Ming Zhu, Yu-Long He, Chang-Hua Zhang

**Affiliations:** Digestive Disease Center, Guangdong Provincial Key Laboratory of Digestive Cancer Research, The Seventh Affiliated Hospital of Sun Yat-Sen University, No. 628 Zhenyuan Road, Shenzhen, 518107 China

**Keywords:** Gastric cancer, NAD, NAMPT, Tumor microenvironment, ATP-adenosine axis, Organoids

## Abstract

**Purpose:**

Nicotinamide adenine dinucleotide (NAD+) is closely related to the pathogenesis of tumors. However, the effect of NAD+ metabolism of gastric cancer (GC) cells on immune cells remains unexplained. We targeted nicotinamide phosphoribosyltransferase (NAMPT), a rate-limiting enzyme in the NAD+ synthesis salvage pathway, to observe its effect in the immune microenvironment.

**Methods:**

NAMPT of GC cell lines was inhibited by using the small molecule inhibitor (FK866) and short hairpin RNA (shRNA). CCK-8 test and flow cytometry were performed to detect cell viability and apoptosis. Immunofluorescence was used to observe changes in mitochondrial membrane potential (MMP).The transfected GC cells (AGS) and patient-derived organoids (PDOs) were cocultured with activated PBMCs, followed by flow cytometric analysis (FCA) for cytokines and inhibitory marker. The level of NAD and ATP of GC cells (AGS & MKN45) was tested combined with NMN and CD39 inhibitor.

**Results:**

Targeting NAD+ by FK866 obviously reduced MMP, which ultimately inhibited proliferation and increased the apoptosis of GC cells. NAMPT silencing reduced intracellular NAD and ATP，further decreased extracellular adenosine. Meawhile, the cytokines of CD8+T cells were significantly increased after cocultured with transfected AGS, and the expression of PD-1 was distinctly decreased. NMN reversed the effect of shNAMPT and enhanced the immunosuppression. Consistent results were obtained by coculturing PBMCs with PDOs.

**Conclusion:**

Restraining the function of NAMPT resulted in the functional improvement of effector CD8+ T cells by decreasing extracellular adenosine levels and inducing apoptosis of GC cells simultaneously. Therefore, this study demonstrates that NAMPT can be an effective target for gastric cancer immunotherapy.

**Supplementary Information:**

The online version contains supplementary material available at 10.1007/s00432-022-04124-9.

## Introduction

Gastric cancer (GC) ranked 5th in global incidence and 4th in mortality, with more than one million new cases and an estimated 769,000 deaths in 2020 (Sung et al. 2021). Despite improvements in technology and equipment, especially the applications of immunotherapy, the global 5-year survival rate for gastric cancer remains unsatisfactory (approximately 25–30%). Of recent concern is the rapidly increasing incidence of gastric cancer in young adults (age < 50 years) in both low and high-risk countries (Arnold et al. 2020; Heer et al. 2020). Thus, the pathogenesis and immune escape mechanism of gastric cancer need to be deeply studied, and it is urgent to develop promising therapies.

The underlying universal feature of tumors is the presence of metabolic reprogramming as well as immune evasion, especially resulting from the interaction of metabolites in the tumor microenvironment (Liberti and Locasale 2016; Katsyuba et al. 2020). Tumors require a greater energy supply for rapid metabolism and proliferation, which leads to enhanced aerobic glycolysis by increasing the uptake of glucose and glutamine, which is known as the “Warburg effect” (Liberti and Locasale 2016). Therefore, more ATP is required to drive aerobic glycolysis in cancer cells. ATP is released during the conversion of nicotinamide adenine dinucleotide (NAD +) to NADH during mitochondrial oxidative phosphorylation (OXPHOS). Thus, NAD+ as a metabolite coenzyme is very important in various physiological processes (Katsyuba et al. 2020).

Consistent with the above, gastric cancer samples have elevated levels of aerobic glycolysis compared to normal tissue (Chen 2010). The intermediates of the tricarboxylic acid cycle (TCA) and the primary five metabolites (α-ketoglutarate, malate, fumarate, succinate, and citrate) are significantly higher in either blood (Song et al. 2012), urine (Hu 2011), or tissue (Chen 2010; Aa et al. 2012; Hirayama et al. 2009; Peng 2011) samples from gastric cancer patients compared to either paraneoplastic or normal tissues, which suggests that gastric cancer cells still use glucose for oxidative phosphorylation (Xiao and Zhou 2017). In addition, although NAD+ is widely present in the nucleus and cytoplasm, its levels in mitochondria are significantly higher than those in the nucleus (Cambronne et al. 2016), so we speculate that the metabolic balance of NAD+ plays a very important role in the tricarboxylic acid cycle of gastric cancer cells, and targeting NAD+ synthesis in gastric cancer cells can significantly alter cellular ATP levels, which is an important source of energy and metabolites released by GC cells into the extracellular compartment (Falzoni et al. 2013). ATP can bind to purine receptors (P2XRs and P2YRs) to promote antitumor immunity (Virgilio 2012). However, in solid tumors, extracellular ATP is rapidly degraded to adenosine (known as the “ATP-adenosine axis”), which binds to adenosine receptors and inhibits immune cell activation and infiltration, and immunosuppression occurs (Allard et al. 2020). Thus, the salvage pathway of NAD synthesis is very important in gastric cancer, which uses cellular catabolites to achieve NAD regeneration and requires fewer synthetic enzymes throughout the process with a high utilization rate (Kennedy et al. 2016).

The pivotal synthesis-limiting enzyme in the salvage pathway of NAD synthesis, nicotinamide phosphateribosyltransferase (NAMPT), is more highly expressed in gastric cancer than in other tumor tissues (Chowdhry et al. 2019). Furthermore, a recent pan-cancer single-cell landscape study of tumor-infiltrating lymphocytes (TILs) revealed that tumor-infiltrating CD8+ T cells in tumors (including GC) were characterized by the emergence of exhausted T cells (Zheng et al. 2021),and one of the main reasons for the poor efficacy of immune checkpoint therapy for gastric cancer is the multiple inhibitory metabolites secreted by tumor cells outside the tumor microenvironment (Li et al. 2019). Previous studies on NAMPT have focused merely on its role in gastric cancer cells (Che 2011), but the immunomodulatory effects of targeted NAMPT therapy on the tumor microenvironment have been less studied. Because the levels of NAD+ and ATP are related to NAMPT, we hypothesize that targeting NAMPT of gastric cancer cells to regulate NAD and ATP metabolism could inhibit cell growth and achieve antitumor effects. Thus, it is urgent to explore whether the levels of NAD+ and ATP generated from gastric cancer cells play a critical role in immune evasion. Herein, we found that regulating NAD levels by targeting NAMPT in gastric cancer cells could alter ATP and extracellular adenosine levels, hence improving the antitumor function of CD8+ T cells in the tumor microenvironment.

## Material and methods

### GC cell lines and activation of PBMCs

Human gastric cancer cell lines and a human normal gastric epithelial cell line (GES-1) were obtained from the American Type Culture Collection (ATCC) or Type Culture Collection of the Chinese Academy of Sciences (Shanghai, China) or received as a gift. The cell lines were maintained in RPMI-1640 or DMEM supplemented with 10% fetal bovine serum, penicillin and streptomycin at 37 °C in a humidified 5% CO_2_ incubator.

PBMCs were obtained from peripheral blood from healthy donors using density gradient centrifugation. PBMCs were collected from the Ficoll interface and washed with PBS. The cells were adjusted to 1 × 10^6^ cells/ml and seeded into six-well plates together with 50 IU human IL-2 (NovoProtein, China) to maintain growth in vitro. Ten microliters of ImmunoCult™ Human CD3/CD28/CD2 T-Cell Activator (Stemcell, Canada) was added to the plates, and the cells were incubated for 72 h in a humidified incubator with 5% CO2 at 37 °C.

### Western blot

Equal amounts of protein samples were separated by 10% SDS‐PAGE and transferred onto PVDF membranes. The membranes were blocked with 5% nonfat milk or 5% BSA in TBST buffer for 1 h at RT, followed by incubation with specific primary antibodies overnight at 4 °C. HRP‐conjugated secondary antibodies (1:1000‐1:3000, CST) were incubated with the PVDF membranes for 1 h at RT. The protein bands were visualized by ECL substrate (Meilunbio, China). The antibodies used were anti‐β‐actin (diluted 1:2000, Proteintech), anti‐NAMPT (WB 1:1000, Proteintech), and anti-GAPDH (diluted 1:1000).

### RNA extraction and quantitative real-time PCR

Total RNA was isolated from cultured cells using TRIzol reagent (Accurate Biology). cDNA was synthesized with Evo M-MLV reverse transcription master mix (Accurate Biology) from 500 ng of RNA from each sample. A SYBR@ Green Pro Taq HS premixed qPCR kit (Accurate Biology) was used for qPCR. Data are shown as ‘2-ΔΔCT’. The primers are listed in Supplementary Table 1.

### RNA interference and transfection

Transfection of GC cells was performed with lentivirus vectors encoding short hairpin RNA (shRNA) specific for NAMPT with green fluorescent protein and puromycin resistance as selective markers. The target sequence of shNAMPT was purchased from VectorBuilder Corp. (Guangzhou, China). Lipofectamine 6000 (Beyotime, China) was used to transduct the shRNA plasmids and Lenti-Easy Packaging System (GeneChem, China) into 293T cells. The viral supernatant was then collected at 48 and 72 h posttransfection. The GC cells and organoids were transfected with culture medium containing appropriate lentiviral particles and polybrene (Beyotime, 1:1000). Stably transfected cells were selected by puromycin (1–2 mg/ml, Boyetime, China) until all live cells fluoresced green.

### Cell viability and apoptosis assay

GC cell viability was measured using a Cell Counting Kit‐8 (Meilunbio, Shanghai, China) after administration of the indicated concentrations of FK866 and incubation for an additional 24, 48, and 72 h. Cells in 24-well plates were washed and incubated with CCK‐8 solution at 37 °C for 1–2 h, and then the optical absorbance value at 450 nm was tested. Cell death of GC cells induced by FK866 (Selleck, Houston, USA) was analyzed with flow cytometry using an Annexin-V/PI assay kit (MultiSciences, China). The cells were finally subjected to CytoFLEX flow cytometry, and the results were analyzed using FlowJo 10 software.

### NAD/NADH quantification

GC cells were seeded into 48-well plates. The amount of total NAD in 1 × 10^5^ GC cells was measured with the NAD/NADH (reduced form of NAD+) quantification kit (Beyotime, Shanghai, China) according to the manufacturer’s instructions using protein concentrations to normalize NAD(H) concentrations (Pmol/μg).

### ATP quantification

GC cells were seeded into 48-well plates (2 × 10^5^). The amount of ATP in the intracellular and supernatant of GC cells was measured with an ATP quantification kit (Beyotime, Shanghai, China) using protein content to normalize ATP concentrations (μM/μg).

### Co-culture of PBMCs with GC cells and organoids

An indirect coculture system was established with transwells with a 0.4 µm pore polyester membrane insert (Corning, USA). Activated T lymphocyte-dominated PBMCs were incubated with GC cells and organoids in 6-well and 12-well plates, respectively. After 48 h of incubation, the PBMCs were collected and stained for flow cytometry.

### Flow cytometric staining

Four hours before staining, 2 µl of cytokine stimulator (Leukocyte Activation Cocktail, with GolgiPlug, BD) per milliliter was added to the culture medium. PBMCs were collected and stained live/dead with 4 µl of Human TruStain FcX™ Blocker per million cells. Next, PBMCs were stained with antibodies against surface antigens for 20 min, and then, the cells were fixed and permeabilized for 30 min. Finally, the cells were stained with antibodies against intracellular cytokines for 20 min. Gating strategy for flow cytometry is shown in supplementary Figure A; antibodies for PBMC staining are listed in Supplementary Table 2.

### Statistical analysis

The results are expressed as the mean ± SEM. Student’s *t* test was used to compare two groups of data. Two-tailed *P* values < 0.05 were considered statistically significant. Two-way ANOVA or one-way ANOVA with Tukey’s multiple comparison test was used for three or more group analyses. All experiments were performed at least three times. All statistical analyses were performed using GraphPad Prism 9 software.

## Results

### NAMPT is upregulated in GC tissues and GC cell lines

**Fig. 1:**
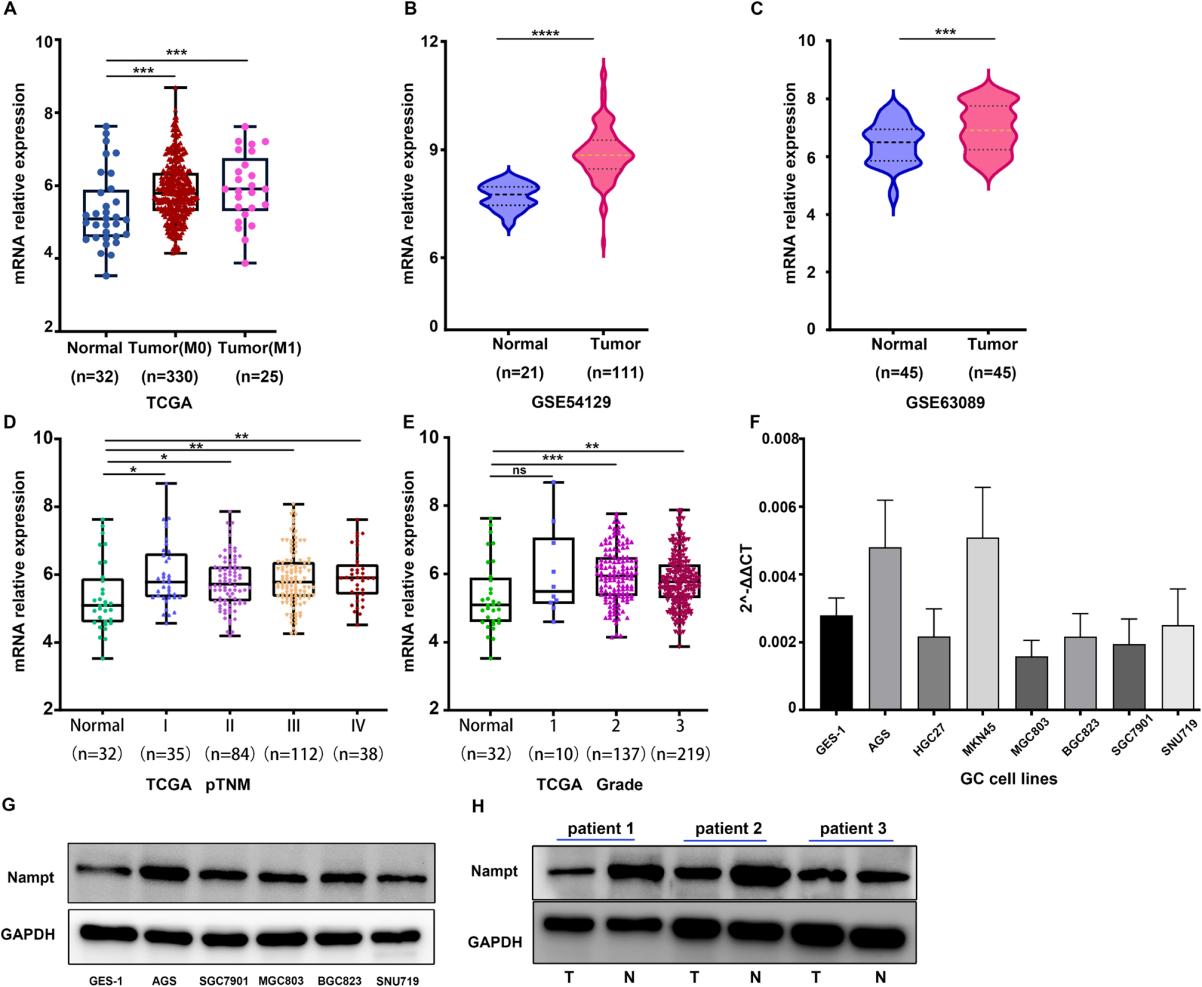
NAMPT is highly expressed in gastric cancer. **a** The mRNA level of NAMPT in gastric cancer from The Cancer Genome Atlas (TCGA) database. **b**, **c** The mRNA level of NAMPT in the unpaired GC tissue dataset GSE54129 and 45 pairs of gastric cancer with adjacent tissue dataset GSE 63089. **d**, **e** The mRNA level of NAMPT in different TNM (I–IV) and tumor grades (1–3) of GC patients downloaded from the TCGA database compared with normal tissues. **f** The mRNA expression of NAMPT in 7 GC cell lines (AGS/HGC27/MKN45/MGC803/BGC823/SGC7901/SNU719) and 1 normal gastric mucosal cell line (GES-1). **g** Western blotting was performed to show the protein level of NAMPT in five GC cell lines and a normal gastric mucosal cell line (GES-1). **h** Western blotting was performed to detect the protein level of NAMPT in three pairs of GC tissues and their paired normal adjacent tissues. All experiments were repeated at least in triplicate (*P < 0.05, **P < 0.01, ***P < 0.001, ****P < 0.0001)

To explore the difference between GC cell lines and GC tissues, we first verified that NAMPT expression was generally higher in gastric cancer tissues and cell lines than in normal or paraneoplastic tissues by Western blot assay (Fig. 1g, h). By exploring the TCGA RNA-seq database, we found that the RNA expression level of NAMPT was also significantly higher than that in normal or paraneoplastic tissues (Fig. 1a). In addition, the expression level of NAMPT was positively correlated with TNM and tumor stage (Fig. 1d, e). We further found that the expression of NAMPT in gastric cancer tumor tissues was significantly higher than that in normal tissues after analyzing the GEO datasets GSE54129 and GSE63089, which was statistically significant (Fig. 1b, c). Although the Western blots showed that NAMPT was generally highly expressed in gastric cancer cell lines (Fig. 1g), there was a large variation in its expression at the mRNA level (Fig. 1f).

### FK866 resulted in a dose and time-dependent reduction in NAD and ATP levels in GC cells

**Fig. 2:**
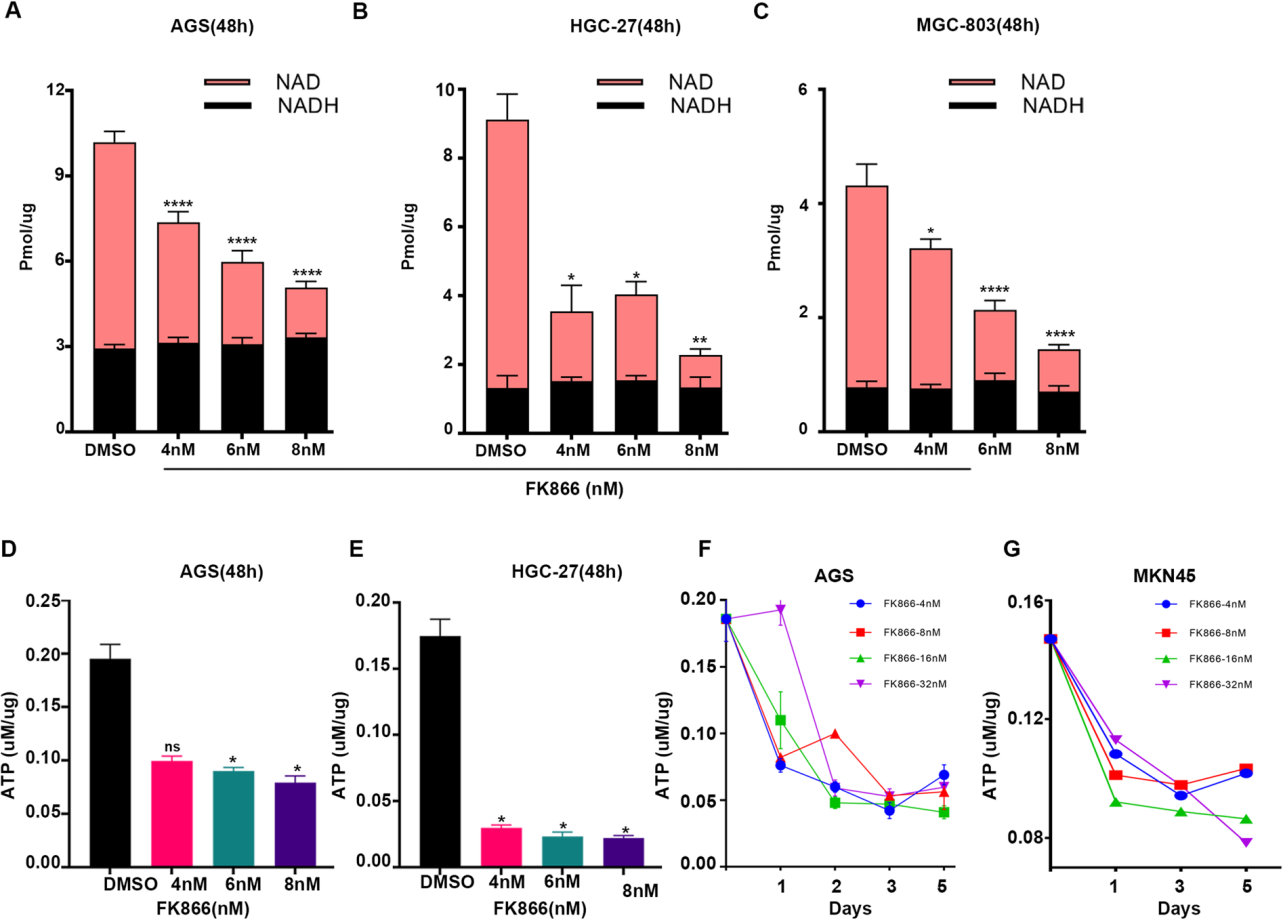
FK866 caused a decrease in NAD and ATP levels in gastric cancer cells, and the extent of the reduction correlated with the increase in FK866 concentration and incubation time. **a**–**c** NAD assay was performed after incubation with AGS, HGC27, and MGC803 cells at three concentrations of FK866. **d**, **e** The ATP levels of AGS and HGC27 cells decreased significantly with increasing FK866 concentration. **f**, **g** An ATP assay was performed after incubation with AGS and MKN45 cells at four concentrations of FK866 for 5 days (*P < 0.05, **P < 0.01, ***P < 0.001, ****P < 0.0001. *ns*: not significant)

We initially treated the gastric cancer cell lines AGS, MGC-803, HGC-27, and MKN45 with the Nampt-specific small molecule inhibitor FK866 (Apo866, Daporinad, TargetMol) at concentrations of 4, 6 and 8 nM for a period of time and found that intracellular NAD(H) decreased after 48 h of incubation, with statistically significant differences (Fig. 2a–c). In addition, HGC27 was more sensitive to the concentration of FK866, and the trend of NAD+ decrease was consistent with that of AGS and MGC-803 cells. No significant changes were observed in the NADH concentration.

Consistent with the reduction in NAD levels, the intracellular ATP of AGS and HGC27 cells treated with FK866 decreased compared to that of the control (DMSO) group at 48 h. As FK866 decreased NAD levels, the intracellular ATP of AGS and HGC27 cells continued to decrease at 48 h, showing a dose-dependent effect (Fig. 2d, e). When treated with FK866 at concentrations of 4, 8, 16 and 32 nM, the intracellular ATP levels continued to decrease over 5 days in AGS and MKN45 cell lines (Fig. 2f, g), and the minimum values of ATP were relatively similar. There was a transient increase in ATP at high concentrations (32 nM) of FK866 in AGS cells, but ATP declined continuously with time. The extent of decrease in ATP in MKN45 at high concentrations (32 nM) was less evident than that at low concentrations, but the reduction in ATP continued just as AGS did. Both cell lines showed a slight rebound of ATP at low concentrations, suggesting a possible drug adaptation (Fig. 2f, g).

### Inhibition of NAMPT targeted the mitochondrial membrane potential and induced apoptosis

**Fig. 3:**
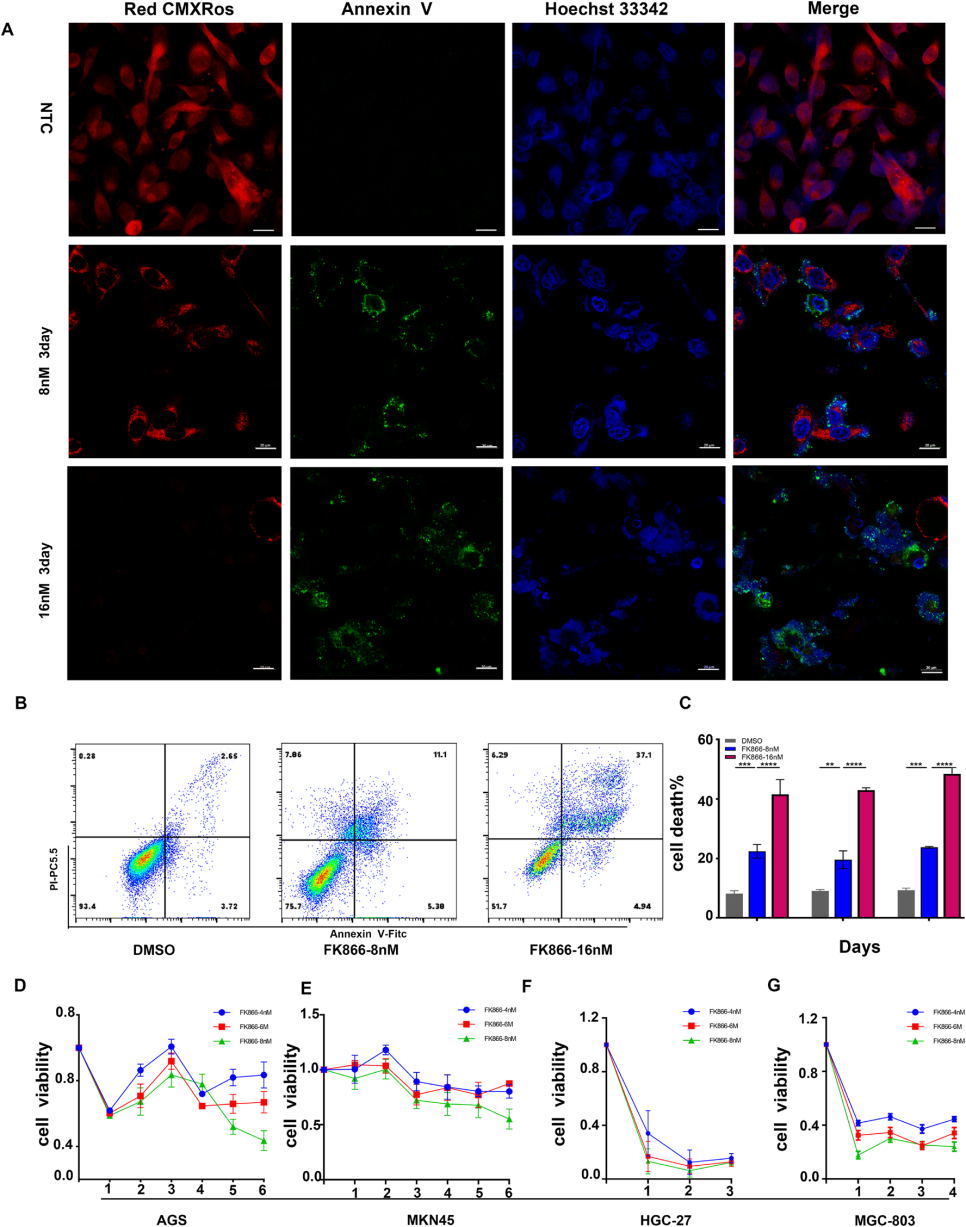
FK866 inhibited ATP production in mitochondria, which resulted in a decline in mitochondrial membrane potential and ultimately induced apoptosis. **a** Incubation of GC cells (AGS) with specific markers for mitochondria after FK866 treatment; MitoTracker CMXRos (red) for mitochondrial membrane potential, Annexin V-FITC (green) for phosphatidylserine exposure of apoptosis. The cell nucleus was stained with Hoechst 33432 (blue). **b**, **c** FCA showed AGS apoptosis induced by FK866 at concentrations of 8 nM and 16 nM. **d**–**g** The CCK-8 assay showed that GC cells (AGS/MKN45/HGC27/MGC803) underwent apoptosis in response to FK866 (**P < 0.01, ***P < 0.001, ****P < 0.0001)

To verify whether NAMPT inhibition by FK866 can suppress NAD levels in mitochondria, we used MitoTracker Red CMXRos to label the MMPs of treated gastric cancer cells and Annexin-V to label apoptotic cells. Using confocal fluorescence microscopy, MitoTracker Red CMXRos could stain the mitochondria to red fluorescence for GC cells (AGS) without any treatment, while the intracellular red fluorescence intensity was significantly decreased in AGS treated with FK866, suggesting that the MMPs were significantly decreased after inhibiting NAD synthesis in mitochondria and the ability to generate ATP was reduced (Fig. 3a). At higher concentrations of FK866 and longer incubation times, the red fluorescence intensity of the mitochondrial membrane potential was lower, while the intensity of green fluorescence on the cell membrane surface gradually increased, and apoptosis occurred. This was further verified by flow cytometry, and a significant trend of apoptosis was found in AGS cells after treatment with FK866 (8 nM and 16 nM) for 2–3 days (Fig. 3b, c). The cell viability decreased gradually with increasing concentration and incubation time, among which the curve of AGS and MKN45 declined slowly and fluctuated (Fig. 3d, e), while HGC27 and MGC803 were more sensitive to the inhibitory effect of FK866 (Fig. 3f, g). Therefore, targeting NAMPT can significantly inhibit NAD+ synthesis in mitochondria, resulting in a reduction in ATP and cell apoptosis.

### NAMPT silencing decreased intracellular NAD and ATP levels in GC cells

**Fig. 4:**
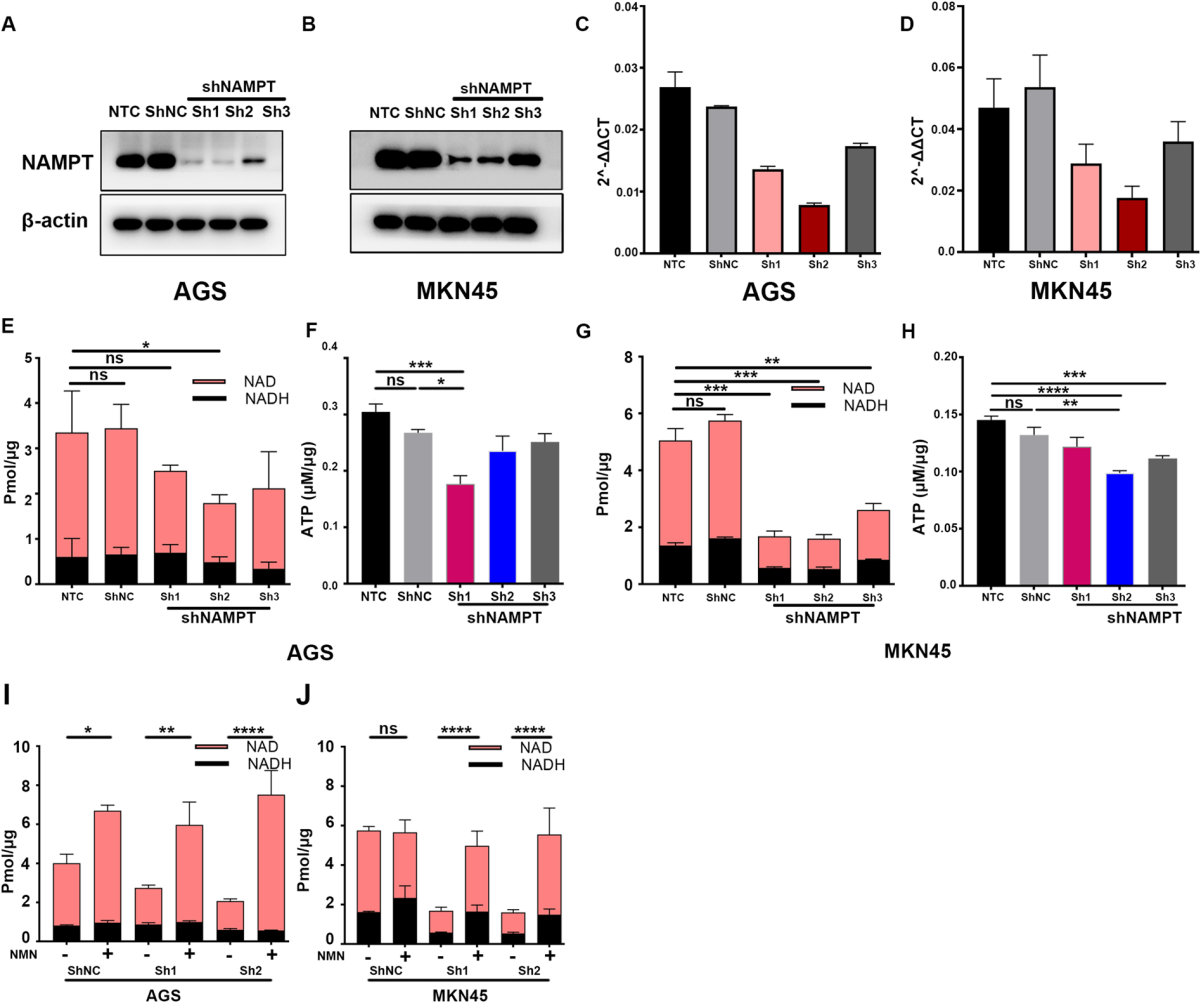
Knockdown of NAMPT by shRNA interference resulted in a reduction in NAD and ATP levels in GC cells. **a**–**d** The knockdown efficiency of NAMPT in GC cell lines (AGS and MKN45) after transfection with shNAMPT lentivirus. **e**–**h** NAD+ and ATP levels in AGS and MKN45 cells after transfection. **i**, **j** NAD levels in lentivirus stably transfected GC cells (AGS/MKN45) after the addition of NMN. All experiments were repeated at least in triplicate (*P < 0.05, **P < 0.01, ***P < 0.001, ****P < 0.0001, *ns* not significant)

We then used RNA interference to silence NAMPT expression in gastric cancer cells. To further study the function of NAMPT in the GC microenvironment, lentiviral transfection was performed to construct stable knockdown GC cell lines (AGS and MKN45). Higher knockdown efficiency was observed (Fig. 4a–d), and further study verified that knockdown of NAMPT in GC cells also decreased the intracellular NAD and ATP levels in AGS (Fig. 4e, f) and MKN45 cells (Fig. 4g, h). In AGS cells, the reduction in ATP was more apparent in the shNAMPT#1 group, while it was more apparent in the shNAMPT#2 group in the MKN45 cell line. Therefore, we used shNAMPT#1 and shNAMPT#2 in the following experiments.

We then added nicotinamide mononucleotide (NMN), the direct precursor for NAD+ synthesis, to the culture medium and observed a significant increase in NAD levels in AGS (Fig. 4i) and MKN45 cells (Fig. 4j), which was more apparent in the knockdown group than in the shControl group.

### NAMPT silencing attenuated immunosuppression of CD8+ T effector cells by GC cell metabolites

To verify the effect of gastric cancer cells on immune cells in the microenvironment, we cocultured the treated gastric cancer cells with activated PBMCs obtained from healthy donors’ peripheral blood to simulate the effect of tumor cell exocrine metabolites on immune cells in the microenvironment in vitro.

**Fig. 5:**
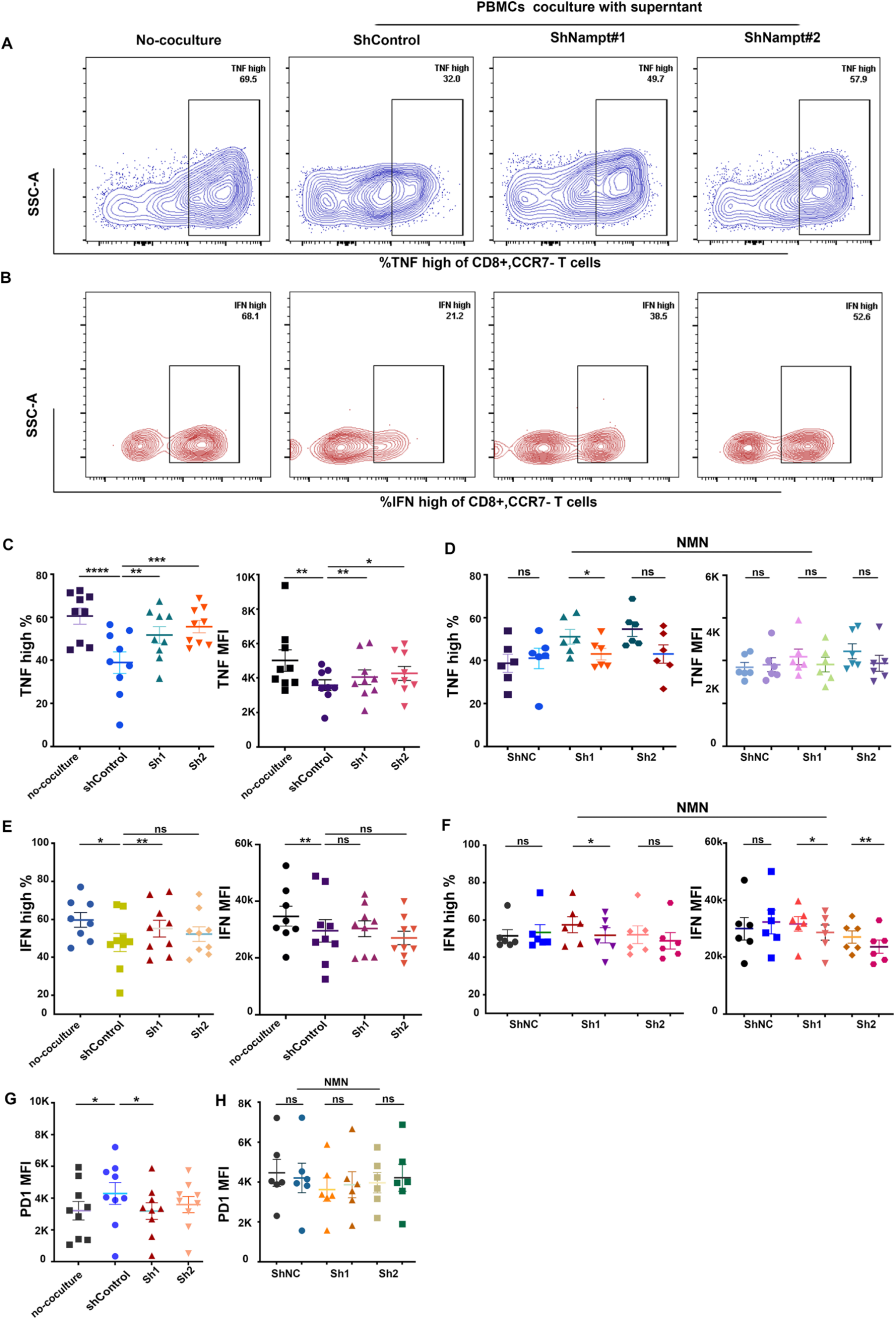
GC cells (AGS) transfected with shNAMPT inhibited the immunosuppressive effect on activated CD8+ T cells in the coculture system of the indicated groups. **a**, **b** Representative flow cytometry staining of the frequencies of TNFα^high^ and IFNγ^high^ CD8+ and CCR7- T cells in the coculture system of the indicated groups. **c**, **d** MFI and frequency of TNFα^high^ CD8+ CCR7-T cells with or without NMN. **e**, **f** MFI and frequency of IFNγ^high^ CD8+ CCR7-T cells with or without NMN. **g**, **h** MFI of PD1 in CD8+ CCR7- T cells with or without NMN. **i**, **j** ATP levels in the culture supernatant of AGS and MKN45 cells treated with or without NMN and SMT in a coculture system. Data are representative of at least three independent experiments (*P < 0.05; **P < 0.01; ***p < 0.001; *ns* not significant)

After coculture with gastric cancer cells via transwell insert, flow cytometric analysis (FCA) showed that the frequency and mean fluorescence intensity (MFI) of cytokines of effector CD8+ T cells (Fig. 5a, b) in the experimental group were significantly decreased compared to the noncultured group. However, although the knockdown group of cocultured GC cells had the same tendency as the shControl group, they had a lower reduction in cytokines: TNFα^high^ % in Sh1 and Sh2 cells (Fig. 5c) and IFNγ^high^ in Sh1 cells (Fig. 5e). The MFI of the immune checkpoint inhibitors of exhaustive CD8+ T cells in the ShControl group was significantly increased compared to that in the noncultured group. Similarly, a slight decrease in exhaustive markers in CD8+ T cells in the shNAMPT group compared to the ShControl group was also observed, of which PD1 was the most representative and statistically significant (Fig. 5g), while TIM3 and LAG3 showed the same tendency but were not statistically significant (Supplementary Figure C).

GC cells treated with NMN were cocultured with PBMCs again, and we found that there was no significant change in the frequency of cytokines (TNFα^high^ and IFNγ^high^) in the ShControl group. However, the frequency of cytokines (TNFα^high^ and IFNγ^high^) in the Sh1 group with NMN was decreased compared to that in the Sh1 group without NMN. The MFI of IFNγ^high^ in the Sh1 and Sh2 groups with NMN was significantly lower than that in the group without NMN (Fig. 5f). There was a decreasing trend in TNFα^high^ MFI, with no significant difference (Fig. 5d). Simultaneously, the exhaustive marker PD1 of CD8+ T cells in the Sh1 groups increased without a significant difference (Fig. 5h).

### NAMPT silencing reversed the dysfunction of CD8+ T effector cells by decreasing extracellular adenosine

**Fig. 6:**
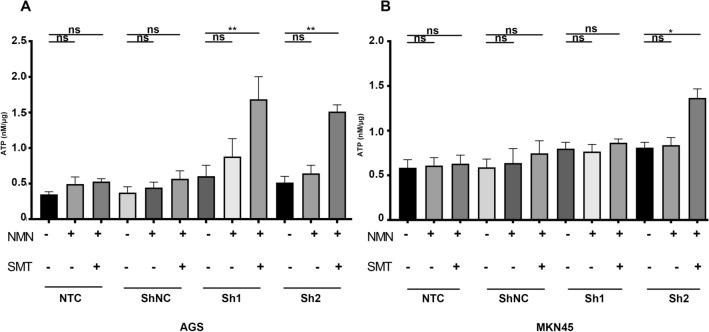
Conversion of ATP to adenosine after NMN supplementation intensifies functional inhibition of CD8+ T cells. **a**, **b**: ATP levels in the culture supernatant of AGS and MKN45 cells treated with or without NMN and SMT in a coculture system. Data are representative of at least three independent experiments (*p < 0.05; **p < 0.01; ***p < 0.001; *ns* not significant)

Based on the results above, we speculated that part of the increased ATP after the addition of NMN released to the extracellular compartment was converted to adenosine by CD39 and CD73 (Cai et al. 2015; Lu 2013). Since the first step of the conversion of ATP to adenosine is catalyzed by CD39 on the membrane surface (ATP to AMP) (Kennedy et al. 2016), we introduced the CD39 inhibitor sodium metatungstate (SMT) to regulate the extracellular ATP and adenosine levels. We found that SMT at a concentration of 8 μm could show the extent of conversion of extracellular ATP to adenosine (Supplementary Figure B). The extracellular supernatant ATP levels in the shNAMPT groups (AGS in Sh1, Sh2, MKN45 in Sh2) with NMN and SMT were significantly higher than those in the shNAMPT groups with NMN only (Fig. 6a, b), suggesting that the extracellular ATP released by the addition of NMN was significantly increased but subsequently converted to adenosine.

### Coculture of transfected GC organoids showed relieved immunosuppressive properties

**Fig. 7:**
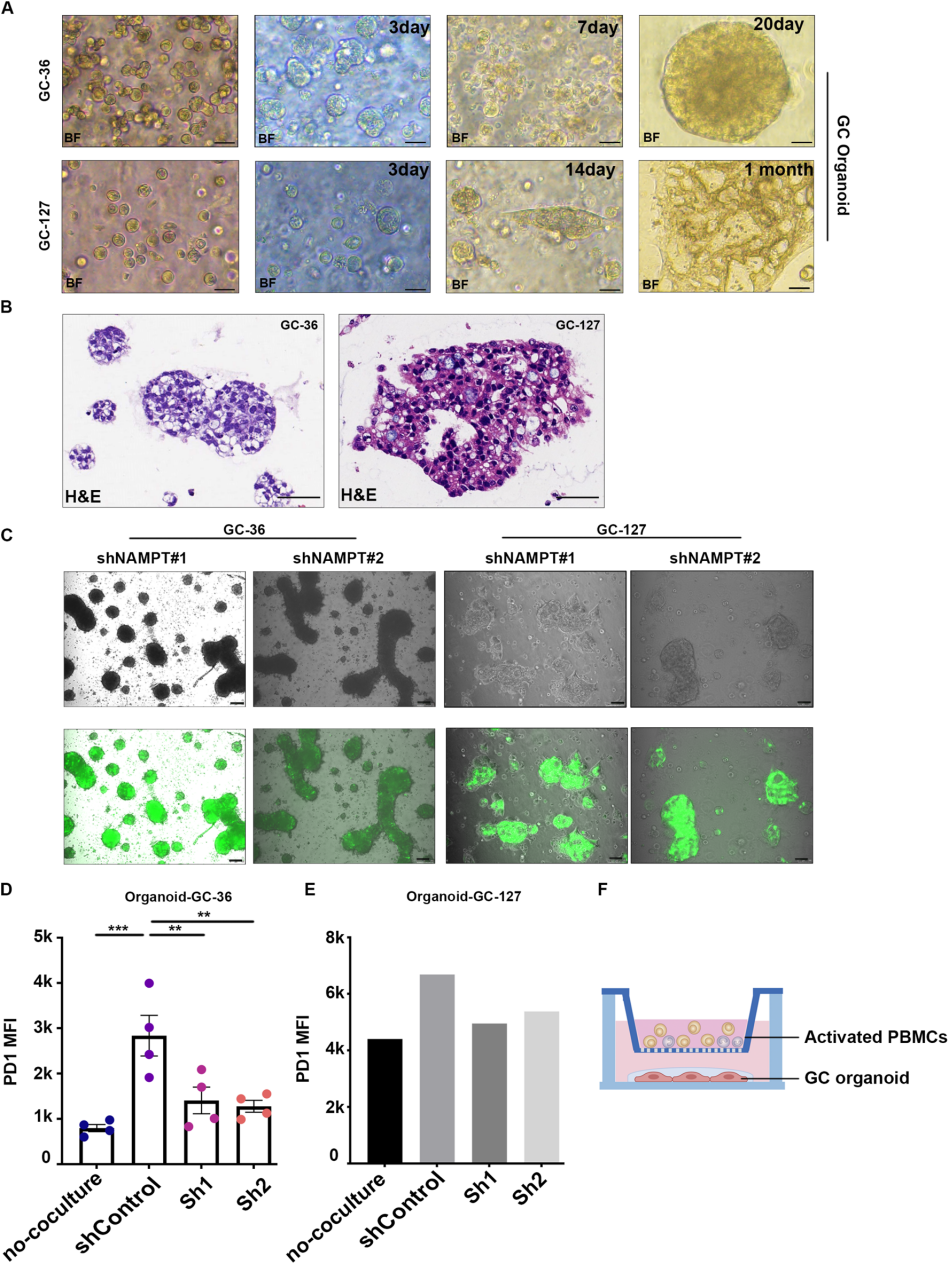
GC organoids transfected with lentivirus-mediated shRNA targeting NAMPT inhibited the immunosuppression of effector CD8+ T cells in vitro. **a** Representative phase-contrast images of GC single cells transformed toward GC organoids in a bright field (scale bars represent 100 μm). **b** H&E staining of GC organoids (scale bars represent 50 μm). **c** Fluorescence identification of the efficiency of shRNA lentivirus transfection in GC organoids (GC36 and 127) (eGFP in green). **d**, **e** MFI of PD1 in CD8+ T cells in a coculture system with GC-Organoid-36/127 by FCA. **f** Establishment of an indirect coculture model of GC organoids and PBMCs in vitro. Data are representative of at least three independent experiments and are shown as the mean ± SEM (*P < 0.05; **P < 0.01; ***P < 0.001; *ns* not significant)

**Fig. 8:**
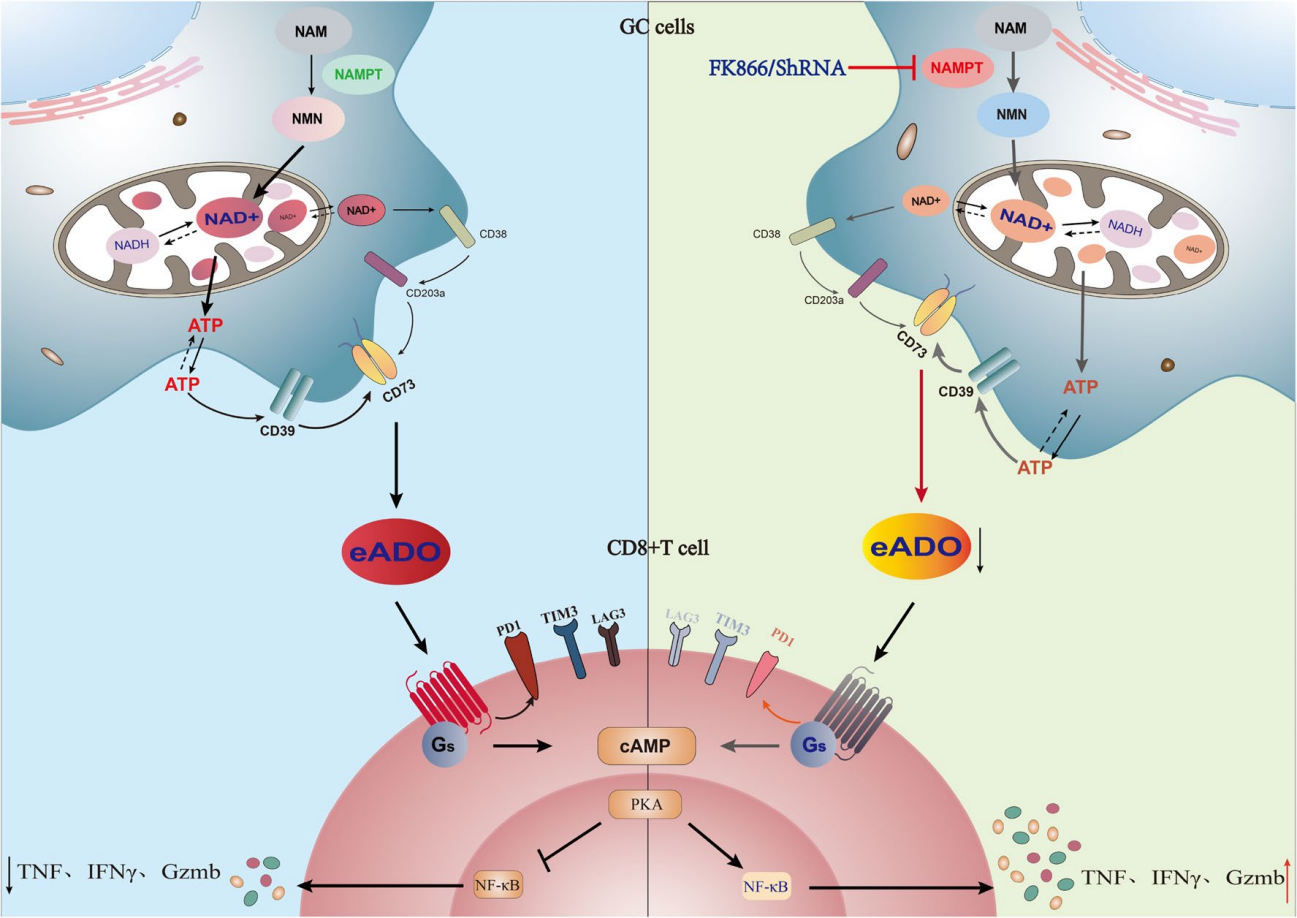
An overview of Schematic diagram of the proposed mechanism for the effect of NAD inhibition on CD8+ T cell in GC TME

Finally, we used GC patient-derived organoids (PDOs) to coculture with activated PBMCs to mimic the immune microenvironment in gastric cancer. We cultured GC organoid strains 36 and 127 from the patients (Fig. 7a, b) and obtained knockdown strains by transfecting organoids with lentivirus (Fig. 7c). After coculturing with PBMCs, FCA showed that, unlike the results of the gastric cancer cell line cocultured with PBMCs, the percentage of cytokines (TNFα^high^ and IFNγ^high^) in the shNAMPT group was not significantly different from that in the ShControl group, but PD1, which represents the exhaustion of CD8+ T cells, was significantly decreased compared with that in the control group (Fig. 7d, e). Meanwhile, the MFI of TIM3 was decreased without a significant difference (Supplementary Figure D), which may be due to the number of experimental groups being insufficient. In conclusion, the results of gastric cancer organoids cocultured with PBMCs indicated that targeting NAMPT in gastric cancer cells could regulate the extracellular adenosine level and inhibit the exhaustion of CD8+ T cells in the immune microenvironment (Fig. 8).

## Discussion

The metabolism of NAD+ is vital in regulating a variety of biological processes, especially in cancers (Chiarugi et al. 2012; Garten et al. 2015). In liver cancer, it has been reported that silencing the expression of NAMPT can exceedingly inhibit PD-L1 immune checkpoint expression (Lv et al. 2021). In TILs, NAMPT-mediated NAD+ production is essential for T-cell activation by generating ATP. Metabolically, NAD+ deficiency leads to defects in aerobic glycolysis and OXPHOS. NAD+ supplementation rescues these defects and enhances the tumor-killing power of T cells (Wang et al. 2021). Thus, NAMPT, which has an important role in regulating NAD+ and ATP synthesis, should also have a role in regulating immunity in the tumor microenvironment. However, previous studies on NAD metabolism have mostly focused on the functional effects of targeting NAMPT on tumor cells alone or only on the metabolic effects on immune cells, without considering the possible overall regulation of the ATP-adenosine axis within the immune microenvironment as a whole. NAD+ metabolism in the immune microenvironment of gastric cancer has been less studied. Research on contact-dependent mechanisms in the tumor microenvironment, such as immunotherapy based on immune checkpoint inhibition or T-cell therapy, is booming, but the efficacy in gastric cancer is unsatisfactory due to the heterogeneity of gastric cancer. Although the infiltration of immune cells exists in solid tumors, multiple factors inhibit immune cells from forming an immunosuppressive tumor microenvironment (Li et al. 2019). Cytotoxic CD8+ T cells are the dominant killer lymphocytes in solid tumors and secrete cytokines, such as IFNγ, TNFα, granzyme B, and perforin. Their activation and proliferation are mainly stimulated by costimulatory and TCR signals. However, a single-cell pancancer study (including GC) found that CD8+ T cells infiltrating the tumor microenvironment mostly presented a noticeably exhausted phenotype (Zheng et al. 2021), and tumor-infiltrated effector CD8+ T cells tended to be exhausted and eventually apoptotic (He et al. 2019). From a spatial perspective, CAFs, TAMs and other stromal cells isolate effector cytotoxic T cells (CTLs) to the edge of the tumor, which facilitates the generation of ‘cold tumors’ and insensitivity to immunotherapy (Vitale et al. 2021). Additionally, tumor cells exert suppressive effects on immune cells by secreting various chemokines and small molecules, leading to exhaustion of effector CD8+ T cells, differentiation of CD4+ T cells to regulator T cells (Tregs), and M2 polarization of macrophages, which, in general, renders the tumor microenvironment immune-suppressive or even cancer-promoting (Li et al. 2019). Therefore, targeting noncontact-dependent immune mechanisms may be a more promising area of research in GC.

Extracellular ATP is maintained at low levels. Due to acidity, hypoxia, and glucose deficiencies in the tumor microenvironment, intracellular ATP is released in large quantities to the extracellular compartment. ATP is then converted to adenosine by CD39 and CD73 (Kennedy et al. 2016), which are highly expressed on the surface of gastric cancer. In addition, NAD can be converted directly to adenosine by the CD38–CD203a–CD73 axis (Ferretti et al. 2019). Adenosine then binds to adenosine receptors at the surface of immune cells (CD8+ T cells mainly through A2aR) as an immune regulator, activating downstream intracellular cyclic AMP (cAMP) signal and inducing immunosuppressive effects (Ohta et al. 2006; Ohta and Sitkovsky 2001) and even T-cell exhaustion(He et al. 2019).

Overall, we hypothesized that adenosine in the extracellular matrix of gastric cancer cells induces CD8+ T cell exhaustion, and that targeting NAMPT in GC cells may reverse the immune suppression of CD8+ T cells in vitro. As previously reported in the literature (Chowdhry et al. 2019; Che 2011), we verified that NAMPT was highly expressed in gastric cancer tissues or cells (Fig. 1). In addition to inhibiting GC cell proliferation, targeting NAMPT resulted in a reduction in ATP levels by suppressing NAD levels (Figs. 2, 3). In the extracellular space, the regulation of ATP-adenosine eventually downregulated immunosuppression on effector CD8+ T cells (Figs. 5, 6).

Unlike direct coculture, we then used an indirect model to study the effect of metabolites on immune cells, excluding the effect of intercellular contact interactions. Consistent with our expectation, CD8+ T-cell exhaustion was alleviated after NAMPT silencing of GC cells in the coculture model, suggesting that immunosuppression could be impeded by targeting NAD metabolism to regulate the ATP-adenosine axis.

Furthermore, it has been reported that GC patient-derived organoids (PDOs), three-dimensional cell clusters cultivated from gastric cancer stem cells and spontaneously organized into organ-like or tissue-like structures (Ohta et al. 2006; ; Vlachogiannis et al. 2018), can retain tumor characteristics and maintain tumor mutational properties for a long time (Ohta and Sitkovsky 2001; Yan et al. (2018); Method of the Year Organoids 2018). Thus, we used GC organoids to coculture with activated PBMCs indirectly to mimic the GC immune microenvironment. As the data showed, although there was no statistically significant change in the percentage of cytokines (data not shown), the T-cell exhaustion marker PD1, the main target of immune checkpoint blockade, was significantly decreased compared with the undisposed group. In addition, the coinhibitory molecule TIM3 showed a slight decreasing tendency, considering that there was no statistically significant difference due to insufficient replications. All of the above results confirmed that the regulation of NAD+ could impede the immunosuppressive status of CD8+ T cells in the immune microenvironment of gastric cancer.

In summary, we conclude that the immunosuppression of effector CD8+ T cells is restrained by inhibiting NAD metabolism through targeting NAMPT in gastric cancer, which eventually reduces the extracellular adenosine level. Inhibition of NAD metabolism may be a target for adjuvant immunotherapy in GC.

## Conclusion

Since the discovery of NAD+, many studies have confirmed its important role as a research target for tumor function and metabolism, focusing on its effects on tumor or immune cells alone. Our study found that by targeting NAD metabolism in GC cells, we can regulate the concentration of adenosine within the microenvironment, which in turn reshaped the metabolism within the microenvironment and reversed the immunosuppressive effects on immune cells, and in particular, the exhaustive status of effector CD8+ T cells, extending the landscape of NAD metabolites on the immune microenvironment to some extent (Fig. 8).

## Limitations

Our experiments were based on gastric cancer cell lines and organoids, which cannot simulate the real situation in vivo. In addition, to investigate the function of NAMPT clearly, we did not use the inhibitor FK866 when tumor cells were cocultured with PBMCs due to the possible effect of FK866 on the metabolism of PBMCs. Therefore, the function of the inhibitor FK866 on immune cells in vivo should be confirmed by further studies.

## Supplementary Information

Below is the link to the electronic supplementary material.

Supplementary file1 (DOCX 4725 KB)

## Abbreviations

GC: Gastric cancer
NAD: Nicotinamide adenine dinucleotide
NAMPT: Nicotinamide phosphoribosyltransferase
shRNA: Short hairpin RNA
FCA: Flow cytometric analysis
OXPHOS: Oxidative phosphorylation
TCA: Tricarboxylic acid cycle
MMP: Mitochondrial membrane potential
TILs: Tumor-infiltrating lymphocytes
TME: Tumor microenvironment
PDOs: Patient-derived organoids
NMN: Nicotinamide mononucleotide
