# Supplementary material for: Targeting NAD metabolism regulates extracellular adenosine levels to improve the cytotoxicity of CD8+ effector T cells in the tumor microenvironment of gastric cancer
Source: J Cancer Res Clin Oncol. 2022 Jul 1;149(7):2743–56. doi: 10.1007/s00432-022-04124-9 (PMC10314862; doi:10.1007/s00432-022-04124-9)
Supplement: Supplementary file 1 — Supplementary file1 (DOCX 4725 KB) [file 432_2022_4124_MOESM1_ESM.docx]

Supplementary Table 1

| **primer** | **sequences** |
| --- | --- |
| hGAPDH-forward primer | CGGAGTCAACGGATTTGGTCGT |
| hGAPDH-reverse primer | TCTCAGCCTTGACGGTGCCA |
| Nampt 1F primer | CGGCAGAAGCCGAGTTCAA |
| Nampt 1R primer | GCTTGTGTTGGGTGGATATTGTT |
| beta-actin(F) primer | GATCATTGCTCCTCCTGAGC |
| beta-actin(R) primer | GGGCCGGACTCGTCATA |

| **Laser line** | | **Antigen** | **Fluorophore** | **Cytoflex LX filter** | | **Company** | | | **Function** |  |
| --- | --- | --- | --- | --- | --- | --- | --- | --- | --- | --- |
| 405nm | CCR7/IFNγ | | BV421 | | V450-PB450 | | BD | Naive T cell marker/cytotoxicity potential | | |
|  | CD45 | | BV510 | | V525-KrO | | BD | Common lymphoid identity | | |
|  | TNFα/CCR7 | | BV605 | | V610 | | BD | Effector function/Naive T cell marker | | |
|  | TIM3 | | BV650 | | V660 | | BD | Co-inhibitory receptor/ immune checkpoint | | |
|  | CD8 | | BV785 | | V763 | | Biolegend | T cell subsets | | |
| 488nm | CD3 | | FITC | | B525-FITC | | BD | T cell subsets | | |
| 561nm | PD1 | | PE-cy^TM^7 | | Y763-PE | | BD | Co-inhibitory receptor/ immune checkpoint | | |
| 638nm | CD4 | | APC | | R660-APC | | BD | T cell subsets | | |
|  | LAG3 | | APC-R700 | | APC-A700 | | BD | Co-inhibitory receptor/ immune checkpoint | | |
|  | LIVE/DEAD | | Zombie fixable viability | | R763-APCA750 | | Biolegend | Exclusion of dead cells | | |

Supplementary Table 2

*Color-coding indicates different lasers and their colors*

Supplementary Figure


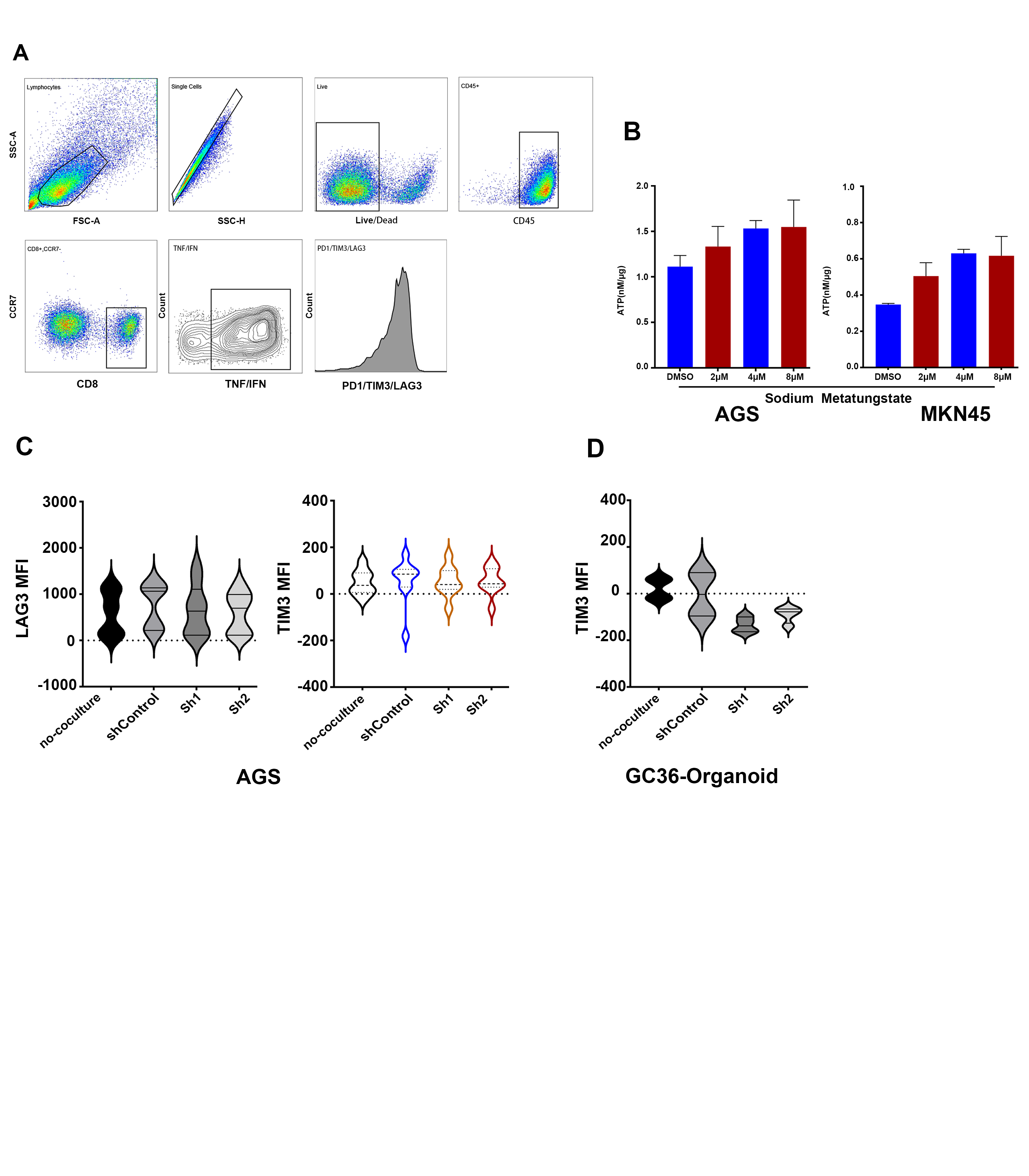


**Fig. Supplementary:**

A: Gating strategy for flow cytometry staining.

B: The ATP levels of supernatant in AGS and MKN45 at three concentrations of SMT for 24h.

C: MFI of LAG3 and TIM3 in CD8+CCR7- T cells after coculture with AGS.

D: MFI of TIM3 in CD8+CCR7- T cells after coculture with GC36 organoid.
